# Editorial: The role of astrocyte in vascular aging

**DOI:** 10.3389/fnagi.2022.961288

**Published:** 2022-08-03

**Authors:** Sen Lin, Feng-Quan Zhou, Jin-Bo Cheng, Xiang-Dong Sun, Gui-Qiong He

**Affiliations:** ^1^Department of Neurology, The Second Affiliated Hospital, Army Medical University, Chongqing, China; ^2^Department of Orthopedic Surgery, Johns Hopkins University School of Medicine, Baltimore, MD, United States; ^3^Department of Neuroscience, Johns Hopkins University School of Medicine, Baltimore, MD, United States; ^4^College of Life and Environmental Sciences, Minzu University of China, Beijing, China; ^5^Key Laboratory of Neuroscience, School of Basic Medical Sciences, Guangzhou Medical University, Guangzhou, China; ^6^Chongqing Key Laboratory of Neurobiology, Department of Anatomy, Institute of Neuroscience, Chongqing Medical University, Chongqing, China

**Keywords:** cerebrovascular aging, BBB, astrocyte, inflammation, astrocyte reprogramming

## Introduction

Aging-induced decline in the function of the cerebrovascular system is one of the most common physiological factors that causes the death of elderly people in the world. Indeed, the incidence of cerebrovascular diseases increases exponentially with age during the later years of people's lives. For people older than 65, hypertension, diabetes, smoking, and aging are the most prominent risk factors for cerebrovascular diseases. Among them, cerebrovascular aging has been largely associated with cellular senescence of the vascular endothelium and the exacerbation of vascular inflammation.

Previous studies have shown that mitochondrial dysfunction, inflammation, loss of proteostasis, genomic instability, epigenetic alterations, progenitor cell exhaustion, and extracellular matrix remodeling are all well-known hallmarks of aging, including cerebrovascular aging. For instance, activation of the ROS-matrix metalloproteases (MMP) axis, an important trigger of tissue aging, could promote the development of cerebral microhemorrhages, which often results in geriatric psychiatric syndromes with cognitive decline and gait disorders. Therefore, better understanding of the cellular mechanisms underlying cerebrovascular aging is of great importance for reducing cerebrovascular-related mortality in an aging population.

This Research Topic aimed at expanding our horizon about how astrocytes contribute to the cerebrovascular aging process and associated diseases. To manage and edit the submitted manuscripts, we invited several guest editors with expertise in cerebrovascular system and/or aging, including Professor Feng-Quan Zhou from Johns Hopkins University School of Medicine, Dr. Jin-Bo Cheng from College of Life and Environmental Sciences at Minzu University of China, Dr. Gui-Qiong He from Chongqing Medical University, Dr. Xiang-Dong Sun from Guangzhou Medical University, and Dr. Sen Lin from Army Medical University. This issue currently includes four papers with various related Research Topics, such as astrocyte reprogramming and a novel method to regulate astrocytes in a neurovascular unit (NVU).

## Astrocytes steer cerebrovascular aging

Increased activities of NAD(P)H (Bagi et al., 2022), cerebrovascular inflammation (Jin et al., 2021), endothelial replicative senescence (Yamazaki et al., 2016), microvascular rarefaction, and pericyte loss (Bell et al., 2010) are major negative contributing factors in brain aging. Astrocytes, the major glial cells in the brain, together with cerebrovascular cells, construct a flexible astrocyte-vasculature unit. Therefore, they have the capacity to control and impact cerebrovascular development, degeneration, diseases, and natural aging. What are the physical clues and chemical factors which astrocytes use to control cerebrovascular aging? One well-known fact is that astrocytic endfeet physically interact closely with cerebrovascular cells and regulate key vascular functions. For instance, aquaporin 4 (AQP4) water channel, the inwardly rectifying K^+^ channel Kir4.1, and the Ca^2+^-dependent K^+^ channel MaxiK are all enriched in astrocyte endfeet, which anchor to the vascular basement membranes *via* the α-β dystroglycan complex (Gondo et al., 2014). Moreover, it has been shown that astrocytes regulate ultra-slow arteriole oscillations *via* stretch-mediated TRPV4-COX-1 feedback (Haidey et al., 2021), which bidirectionally communicate between arterioles and astrocyte endfeet to regulate oscillatory microvasculature activity, providing an unambiguous physical clue for the physical relationship between astrocytes and the cerebrovascular system. As a result, normal brain aging is marked by vascular deficits induced by astrocytic endfeet retraction or separation from blood vessels.

For chemical factors, astrocytes are capable of sensing neuronal activity changes *via* glutamatergic and purinergic receptors, which lead to increased Ca^2+^ levels in endfeet and the release of vasoactive molecules, such as sonic hedgehog (Shh), angiopoietin, and glial-derived neurotrophic factor. These proteins then act on pericytes to modulate blood vessel diameter (Mulligan and MacVicar, 2004; Mishra et al., 2016). In addition, a review article (Han et al.) titled “*The Important Double-Edged Role of Astrocytes in Neurovascular Unit After Ischemic Stroke*” published in a recent organized topic, “*The Role of Astrocyte in Vascular Aging*,” summarizes how astrocytes play an important role in the regulation of blood–brain barrier (BBB) permeability. Moreover, interactions between astrocytes and endothelial progenitor cells may mediate neurovascular remodeling after stroke (Han et al.). Thus, astrocytes not only play essential roles in protecting neurons by releasing neurotrophic factors, but can also be detrimental to the pathological process of cerebral ischemia by activating microglia and disrupting the BBB.

## Perspective of attenuation of cerebrovascular aging by astrocytes

**Figure 1:**
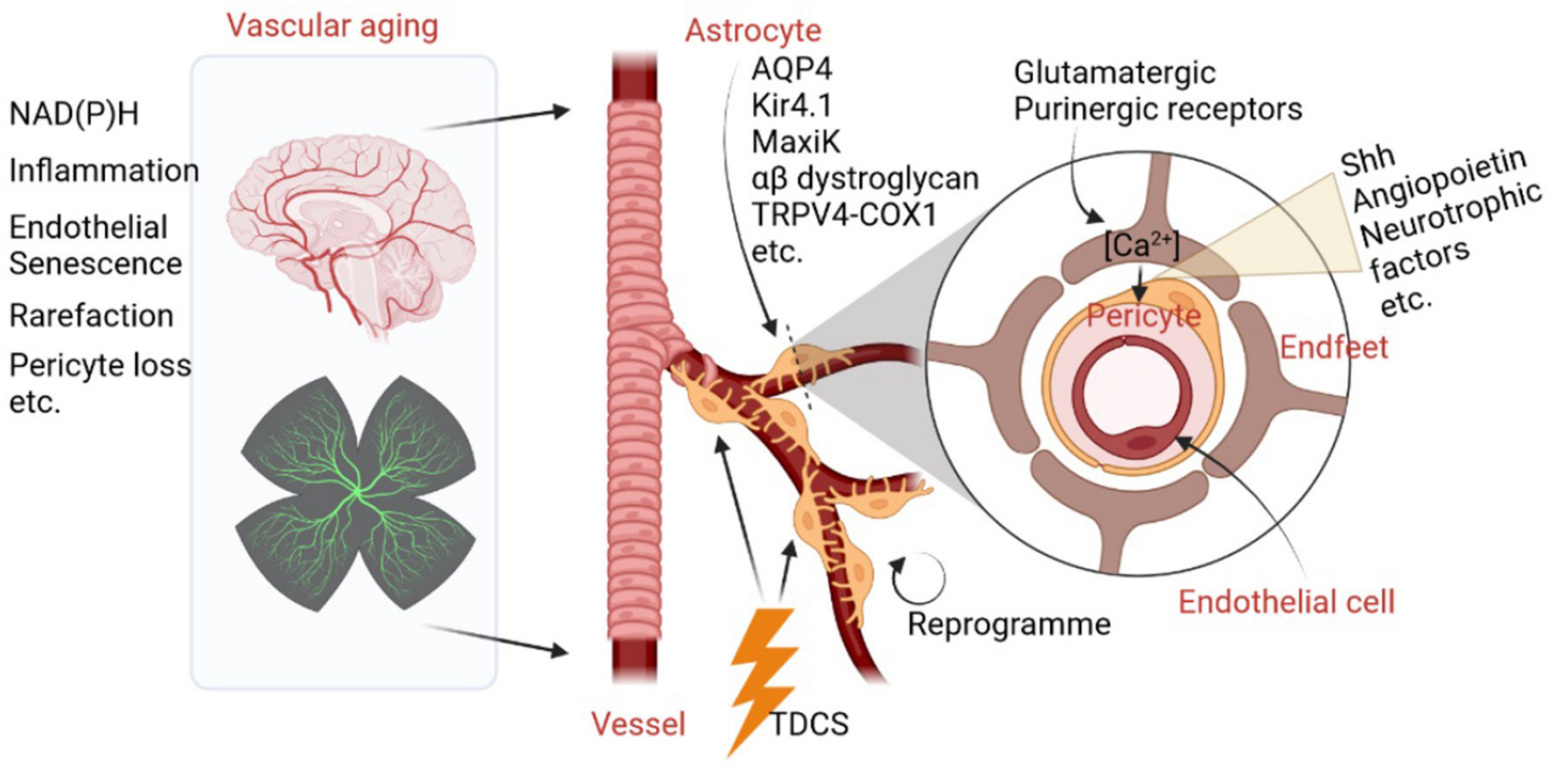
Schematic diagram summarizes the essential roles of astrocytes on vasculature aging.

Because astrocytes play pivotal roles in the process of vascular aging and ischemic stroke, a variety of drugs targeting astrocytes and astrocyte reprogramming methods have been investigated to reduce cerebrovascular-related CNS damage. In the current topic, Han et al. summarized a series of small molecules that could potentially target astrocytes to alleviate cerebral injury and promote neurogenesis and functional recovery. Wen and colleagues reported that in mice with preclinical Alzheimer's Disease, Transcranial Direct Current Stimulation (TDCS) method can be used to alleviate NVU dysfunction (Luo et al.) *via* detecting different levels of important markers of NVU and BBB integrity. Therefore, TDCS is considered to be a novel method that benefits astrocytes and blood vessels. In addition, Peng et al. have reviewed how astrocytes could be reprogrammed by transcription factors, microRNAs, and small molecules to have beneficial roles in stroke. Therefore, astrocyte reprogramming, either trans-differentiation or de-differentiations, could not only provide newly generated neurons after neural injury, but also contribute to astrocyte-vascular unit reconstitution. As a result, such a strategy may shed light on delaying brain aging and treating ischemia stroke through coordinated angiogenesis and neurogenesis. Collectively, cerebrovascular aging, NVU homeostasis, and dysfunction are tightly linked with astrocytes. The essential roles of astrocytes on vasculature aging are shown in Figure 1.

## Author contributions

SL contributed to organize the topic review, invited guest editors, and wrote the manuscript. F-QZ, J-BC, X-DS, and G-QH contributed to invited reviewer and review the manuscripts. F-QZ polished and revised the editorial. All authors contributed to the article and approved the submitted version.

## Funding

SL was supported by the National Natural Science Foundation of China (No. 82071392).

## Conflict of interest

The authors declare that the research was conducted in the absence of any commercial or financial relationships that could be construed as a potential conflict of interest.

## Publisher's note

All claims expressed in this article are solely those of the authors and do not necessarily represent those of their affiliated organizations, or those of the publisher, the editors and the reviewers. Any product that may be evaluated in this article, or claim that may be made by its manufacturer, is not guaranteed or endorsed by the publisher.
